# Genetic heteroscedastic models for ordinal traits: application to sheep litter size

**DOI:** 10.1186/s12711-016-0202-4

**Published:** 2016-04-01

**Authors:** Samira Fathallah, Loys Bodin, Ingrid David

**Affiliations:** GenPhySE, Université de Toulouse, INRA, INPT, INP-ENVT, Castanet-Tolosan, France

## Abstract

**Background:**

Classical genetic canalization models, which accommodate the mean and variance of a trait separately, provide a flexible approach to take heteroscedasticity for continuous traits into account. However, this model is not appropriate for discrete traits. The aim of this work was to propose heteroscedastic threshold models suitable for the genetic analysis of ordinal data.

**Methods:**

In order to first fit the mean and variance of ordinal traits separately, we extended the classical threshold model (TM) for discrete data by introducing non-genetic and genetic factors of heterogeneity on the variance of its underlying variable, which leads to a homothetic threshold model HTM and its alternative parameterization HTM’ in which the thresholds of different individuals are linked by a homothetic-translation. Relaxing the constraint between the thresholds led us to propose an independent threshold model ITM that was more flexible than HTM’ but required the estimation of more parameters. TM, HTM and ITM models were applied to study 19,671 records on litter size in Romane sheep.

**Results:**

Both HTM and ITM were able to disentangle the link between the mean and variance that holds in the classical homoscedastic threshold model. The results obtained for the litter size of Romane ewes showed that the data was best fitted with HTM compared to ITM and TM. The correlations between the observed and predicted variances were equal to 0.6 and 0.2 for HTM and TM, respectively. These analyses showed the existence of a genetic component for the heterogeneity of litter size in sheep that was taken into account in HTM.

**Conclusions:**

HTM is the most suitable model to study the variability of litter size in sheep. It accommodates both the mean and variance separately while requiring the estimation of only a few parameters.

## Background

Robustness can be viewed as the ability to maintain a stable phenotype regardless of the environmental conditions. In line with this approach, Scheiner and Lyman [1] proposed a model which considers that the expression of a trait is controlled by two sets of genes. One set controls the level of the performance and the other set controls the environmental variability of the trait. Various authors developed heteroscedastic models (i.e. canalization models) to jointly estimate the influence of various factors on both the mean and the variability of a trait [2–5].

These models were implemented with frequentist [6, 7] or Bayesian methods [8, 9], and applied to a large variety of continuous traits (birth weight of piglets, birth weight of rabbits, weight of chickens, etc.).

The methods described above mainly target continuous traits. Theoretically, these linear models do not analyze discrete traits adequately because of their non-normal distributions. However, some previous empirical studies showed that the performance of linear and non-linear models was similar in terms of goodness-of-fit and predictive ability [10, 11] when analyzing the level of discrete livestock data. However, it is not known whether these properties are still true when dealing with the variability of discrete data.

The main objective of this research was to propose new models that take the heterogeneous individual variability of ordinal traits into account using derived threshold models. The models are described consecutively in the “Methods” section. “Heteroscedastic extensions of the threshold model” are presented in the first subsection, and a “Numerical application” of these models to litter size in Romane sheep is presented in the second subsection.

## Methods

### Heteroscedastic models for ordinal traits

#### Homothetic threshold models for ordinal traits

A simple way of studying environmental sensitivity of an ordinal trait is to combine the threshold model (TM) [12] (appropriate for the study of ordinal traits) and the canalization model (appropriate for continuous traits) [13]. Thus, heteroscedasticity is considered under the liability threshold model and we call this new model the homothetic threshold model or HTM:

$${\mathbf{t}} = \left( {0,1,\tau_{3} , \ldots ,\tau_{n - 1} } \right);\quad \left. \varvec{l} \right|{\mathbf{u}}, {\varvec{\upnu}} \sim {\text{N}}\left( {1\mu + {\mathbf{u}},f^{2} \left( {\eta ,{\varvec{\upnu}}} \right)} \right),$$

where $\varvec{l}$ is the normally distributed liability and ${\mathbf{t}}$ is a set of thresholds, which transform this liability into the observed discrete variable $y$ in $n$ categories, $\mu$ is the overall mean, ${\mathbf{u}}$ the vector of additive genetic effects that affect the mean of the liability, $\eta$ is the overall mean and ${\varvec{\upnu}}$ is the vector of additive genetic effects that affect the variability of the liability. Several functions $f$ have been proposed in the literature (exponential, linear or square root functions [3]). The vectors of genetic values are assumed to be Gaussian:

$$\left( {\begin{array}{c} {\mathbf{u}} \\ {\varvec{\upnu}} \\ \end{array} } \right)\sim {\text{N}}\left( {\left[ {\begin{array}{c} 0 \\ 0 \\ \end{array} } \right], {\mathbf{G}} \otimes {\mathbf{A}}} \right),$$

where $\otimes$ denotes the Kronecker product, ${\mathbf{A}}$ is the known additive genetic relationship matrix and ${\mathbf{G}}$ the genetic matrix $$\left[ {\begin{array}{cc} {\sigma_{u}^{2} } & {\rho \sigma_{u} \sigma_{\nu } } \\ {\rho \sigma_{u} \sigma_{\nu } } & {\sigma_{\nu }^{2} } \\ \end{array} } \right]$$, where $\sigma_{u}^{2}$ and $\sigma_{\nu }^{2}$ are the variances of the additive genetic effects that affect the mean and the variability of the liability, and $\rho$ their coefficient of correlation.

A fully equivalent parameterization of HTM can be written. This parameterization or HTM’ model is reached by fixing the parameters of the liability and considering that the matrix of thresholds ${\mathbf{T}'}$ is affected by environmental and genetic effects i.e.:

$$\begin{aligned} {\mathbf{T}}' & = \left( {\begin{array}{cccc} {\tau_{11}^{'} } & {\tau_{21}^{'} } & \ldots & {\tau_{{\left( {n - 1} \right)1}}^{'} } \\ {\tau_{1i}^{'} } & {\tau_{2i}^{'} } & \ldots & {\tau_{{\left( {n - 1} \right)i}}^{'} } \\ \cdot & \cdot & & \cdot \\ {\tau_{1N}^{'} } & {\tau_{2N}^{'} } & \ldots & {\tau_{{\left( {n - 1} \right)N}}^{'} } \\ \end{array} } \right) \\ & = \left( {\begin{array}{cccc} {{\mathbf{t}}_{1}^{'} = {\mathbf{\mu^{\prime}}} + u_{1}^{'} \mathbf1^{{\mathbf{T}}} + {\mathbf{v}}_{1}^{'} } \\ {{\mathbf{t}}_{i}^{'} = {\mathbf{\mu^{\prime}}} + u_{i}^{'} \mathbf1^{{\mathbf{T}}} + {\mathbf{v}}_{i}^{'} } \\ \cdot \\ {{\mathbf{t}}_{N}^{'} = {\mathbf{\mu^{\prime}}} + u_{N}^{'} \mathbf1^{{\mathbf{T}}} + {\mathbf{v}}_{N}^{'} } \\ \end{array} } \right) \\ & = \left( {\varvec{\tau}_{1}^{'} ,\varvec{\tau}_{2}^{'} ,\varvec{\tau}_{3}^{'} , \ldots ,\varvec{\tau}_{{\left( {n - 1} \right)N}}^{'} } \right);\quad \varvec{l} \sim {\text{N}}\left( {0,1} \right). \\ \end{aligned}$$

Under this parameterization, the vector of thresholds ${\mathbf{t}}_{i}^{'}$ is specific to each individual $i$, with ${\mathbf{t}}_{i}^{'} = {\mathbf{\mu^{\prime}}} + u_{i}^{'} \mathbf{1}^{{\mathbf{T}}} + {\mathbf{v}}_{i}^{'}$ where ${\mathbf{\mu^{\prime}}}$ is an overall set of $n - 1$ thresholds, $u_{i}^{'}$ a subject-specific deviation from this overall set and ${\mathbf{v}}_{i}^{'}$ a vector of size $n - 1$ that reflects the specific deviation of the subject threshold from this overall set with:

$$\left[ {\begin{array}{c} {{\mathbf{u}}^{{\prime }} } \\ {{\mathbf{v}}_{.1}^{{\prime }} } \\ {{\mathbf{v}}_{.2}^{{\prime }} } \\ {{\mathbf{v}}_{.3}^{{\prime }} } \\ \\ {{\mathbf{v}}_{.n - 1}^{{\prime }} } \\ \end{array} } \right] \sim {\text{N}}\left( {0, {\mathbf{G}}^{{\prime }} \otimes {\mathbf{A}}} \right),$$

where

$${\mathbf{G}}^{\prime } = \left[ {\begin{array}{*{20}c} {\sigma _{{u^{\prime } }}^{2} {\text{ }}} & 0 & {\sigma _{{u^{\prime } ,\nu ^{\prime } }} \left( {\mu _{2}^{\prime } - \mu _{1}^{\prime } } \right)} & {\sigma _{{u^{\prime } ,\nu ^{\prime } }} \left( {\mu _{3}^{\prime } - \mu _{1}^{\prime } } \right)} & {\sigma _{{u^{\prime } ,\nu ^{\prime } }} \left( {\mu _{{n - 1}}^{\prime } - \mu _{1}^{\prime } } \right)} \\ 0 & 0 & 0 & 0 & 0 \\ {} & {} & {\left( {\mu _{2}^{\prime } - \mu _{1}^{\prime } } \right)^{2} \sigma _{{\nu ^{\prime } }}^{2} } & {\left( {\mu _{2}^{\prime } - \mu _{1}^{\prime } } \right)\left( {\mu _{3}^{\prime } - \mu _{1}^{\prime } } \right)\sigma _{{\nu ^{\prime } }}^{2} } & {\left( {\mu _{2}^{\prime } - \mu _{1}^{\prime } } \right)\left( {\mu _{{n - 1}}^{\prime } - \mu _{1}^{\prime } } \right)\sigma _{{\nu ^{\prime } }}^{2} } \\ {\text{ }} & {} & {} & {\left( {\mu _{3}^{\prime } - \mu _{1}^{\prime } } \right)^{2} \sigma _{{\nu ^{\prime } }}^{2} } & {\left( {\mu _{3}^{\prime } - \mu _{1}^{\prime } } \right)\left( {\mu _{{n - 1}}^{\prime } - \pi _{1}^{\prime } } \right)\sigma _{{\nu ^{\prime } }}^{2} } \\ {\text{ }} & {} & {} & {} & {\left( {\mu _{{n - 1}}^{\prime } - \mu _{1}^{\prime } } \right)^{2} \sigma _{{\nu ^{\prime } }}^{2} } \\ {\text{ }} & {} & {} & {\text{ }} & {} \\ \end{array} } \right]$$

Under this parameterization, the correlation between $$\varvec{\tau}_{1.}^{'}$$ and $$\varvec{\tau}_{2.}^{'}$$ is set to 1 and the transformation of $${\mathbf{t}}_{i}^{'}$$ to $${\mathbf{t}}_{i'}^{'}$$ (from individuals $$i$$ to $$i')$$ is not a translation as in TM but a homothetic translation, which means that the ratio $$R = \left( {\tau_{{k_{3} i}}^{'} - \tau_{{k_{1} i}}^{'} } \right)/\left( {\tau_{{k_{2} i}}^{'} - \tau_{{k_{1} i}}^{'} } \right)$$ is constant for the whole population i.e.:

$$\begin{aligned} & \left( {\tau_{{k_{3} i}}^{'} - \tau_{{k_{1} i}}^{'} } \right)/\left( {\tau_{{k_{2} i}}^{'} - \tau_{{k_{1} i}}^{'} } \right) \\ & \quad = \left( {\tau_{{k_{3} i^{\prime}}}^{'} - \tau_{{k_{1} i^{\prime}}}^{'} } \right)/\left( {\tau_{{k_{2} i^{\prime}}}^{'} - \tau_{{k_{1} i^{\prime}}}^{'} } \right), \quad \forall k_{1} , k_{2} , k_{3} \\ & \quad = \left( {\mu_{{k_{3} }}^{'} - \mu_{{k_{1} }}^{'} } \right)/\left( {\mu_{{k_{2} }}^{'} - \mu_{{k_{1} }}^{'} } \right). \\ \end{aligned}$$

HTM and HTM’ are equivalent models with the same number of parameters ($n + 1$) being estimated. The relationship between ${\mathbf{t}}_{i}^{'}$ and ${\mathbf{t}}$ is:

$${\mathbf{t}}_{i}^{'} = \frac{{{\mathbf{t}} - \left( {\mu + u_{i} } \right)\mathbf{1}^{{\mathbf{T}}} }}{{f\left( {\eta ,\nu_{i} } \right)}}.$$

The estimations obtained with HTM’ can be transformed into the estimations that would have been obtained with HTM. Overall, in the case of a linear function $f$, the approach produces:

$$\sigma_{\nu }^{2} = \frac{{\sigma_{{{{\upnu^{\prime}}}}}^{2} }}{{\left( {\mu_{2}^{'} - \mu_{1}^{'} } \right)^{2} }},$$

whereas for the other parameter we have:

$$\sigma_{u}^{2} = \frac{1}{{\left( {\mu_{2}^{'} - \mu_{1}^{'} } \right)^{2} }}\left( {\left( {\mu_{1}^{'} } \right)^{2} \sigma_{{\nu^{\prime}}}^{2} + \sigma_{{u^{\prime}}}^{2} + \sigma_{{\nu^{\prime}}}^{2} \sigma_{{u^{\prime}}}^{2} + \left( {\sigma_{{\nu^{\prime},u^{\prime}}} } \right)^{2} - 2\mu_{1}^{'} \sigma_{{\nu^{\prime},u^{\prime}}} } \right).$$

Finally, the genetic correlation $\rho$ is:

$$\rho = \frac{1}{{\left( {\mu_{2}^{'} - \mu_{1}^{'} } \right)^{2} }}\left( {\frac{{\sigma_{{\nu^{\prime},u^{\prime}}} - \mu_{1}^{'} \sigma_{{\nu^{\prime}}}^{2} }}{{\sigma_{u} \sigma_{\nu } }}} \right).$$

These formulas are detailed in “Appendix 1”. The heritability of the trait on the liability scale is estimated by the ratio of the animal variance that acts on the mean to the total variance of the underlying variable i.e.:

$$h_{1}^{2} = \frac{{\sigma_{u}^{2} }}{{\sigma_{u}^{2} + f^{2} \left( {\eta ,\sigma_{\nu } } \right)}}.$$

For instance in the case of a linear function $f$:

$$h_{1}^{2} = \frac{{\sigma_{u}^{2} }}{{\sigma_{u}^{2} + \eta^{2} + \sigma_{\nu }^{2} }}.$$

#### Extension of the homothetic threshold model into the independent threshold model

A straightforward extension of the above HTM’ model can also be considered and consists in releasing the constraint of the homothetic-translation link between the thresholds of the individuals. The model therefore becomes an independent threshold model ITM, i.e.:

$$\begin{aligned} {\mathbf{T}} & = \left( {\begin{array}{cccc} {\tau_{11}^{ } } & {\tau_{21}^{ } } & \ldots & {\tau_{{\left( {n - 1} \right)1}}^{ } } \\ {\tau_{1i}^{ } } & {\tau_{2i}^{ } } & \ldots & {\tau_{{\left( {n - 1} \right)i}}^{ } } \\ \cdot & \cdot & & \cdot \\ {\tau_{1N}^{ } } & {\tau_{2N}^{ } } & \ldots & {\tau_{{\left( {n - 1} \right)N}}^{ } } \\ \end{array} } \right) = \left( {\begin{array}{c} {{\mathbf{t}}_{1}^{ } = {{\mu}}^{ } + {\mathbf{v}}_{1}^{ } } \\ {{\mathbf{t}}_{i}^{ } = {{\mu}}^{ } + {\mathbf{v}}_{i}^{ } } \\ \cdot \\ {{\mathbf{t}}_{N}^{ } = {{\mu}}^{ } + {\mathbf{v}}_{N}^{ } } \\ \end{array} } \right) \\ & = \left( {\varvec{\tau}_{1.}^{ } ,\varvec{\tau}_{2.}^{ } ,\varvec{\tau}_{3.}^{ } , \ldots ,\varvec{\tau}_{{\left( {n - 1} \right).}}^{ } } \right);\quad \varvec{l} \sim {\text{N}}\left( {0,1} \right), \\ \end{aligned}$$

where ${\mathbf{t}}_{i}^{ } = {{\mu}}^{ } + {\mathbf{v}}_{i}^{ }$ with ${{\mu}}$ being an overall set of $n - 1$ thresholds, and ${\mathbf{v}}_{i}^{ }$ a vector of size $n - 1$, which reflects the specific deviation of the subject threshold from this overall set with:

$$\left[ {\begin{array}{c} {{\mathbf{v}}_{.1}^{ } } \\ {{\mathbf{v}}_{.j}^{ } } \\ \vdots \\ {{\mathbf{v}}_{.n - 1}^{ } } \\ \end{array} } \right] \sim {\text{N}}\left[ {0,\left[ {\begin{array}{cccc} {\sigma_{{\nu_{.1} }}^{2} } & {\sigma_{{\nu_{.1} \nu_{.j} }} } & \cdots & {\sigma_{{\nu_{.1} \nu_{.n - 1} }} } \\ & {\sigma_{{\nu_{.j} }}^{2} } & \cdots & {\sigma_{{\nu_{.j} \nu_{.n - 1} }} } \\ & & \ddots & \vdots \\ & & & {\sigma_{{\nu_{.n - 1} }}^{2} } \\ \end{array} } \right] \otimes {\mathbf{A}}} \right].$$

The total number of parameters in this model is $\left( {n + 2} \right)\left( {n - 1} \right)/2$. It should be noted that ITM cannot be parameterized like HTM using a fixed set of thresholds and a model on the liability $l$. In this model, fixing the thresholds to modify the parameterization would imply that the underlying variable would no longer be Gaussian.

Due to the lack of constraints between the thresholds (except for the obvious relationship $- \infty < \tau_{1.} < \tau_{2.} < \cdots < \tau_{n - 1.} < \infty$), ITM fits a much larger range of situations than a homothetic threshold model, which assumes a strong constraint either on the homothetic translation relationship between thresholds (HTM’) or on the normality of the liability (HTM).

Interestingly, we found that another parameterization of ITM is a constrained multiple-trait model MTM (Appendix 2). Indeed, each observation of the ordinal trait can be considered as the result of $n$ exclusive binary traits:

$$1_{k} \left( y \right) : = \left\{ {\begin{array}{ll} 1 & \quad \hbox{if} \,y = k/k = 1 \ldots n \\ 0 &\quad \hbox{otherwise} \\ \end{array} } \right.$$

The observed variable $y$ can thus be transformed into $n$ indicator functions which can be analyzed using a multivariate binary threshold model. Only $n - 1$ indicator functions need to be considered in this MTM since the value of one of the indicator functions is fixed conditionally to the others. Each sub-model $k, k \in \left[ {1, n - 1} \right]$ can be written as ${\text{MTM}}_{k}$:

$$\left. {\tau_{k} } \right|u_{k} ;\quad l_{k} \sim{\text{N}}\left( {0,1} \right).$$

According to this model, the probability that an individual might achieve the observation $k$ or that the *k*^t^^h^ indicator function is equal to 1 is:

$$\pi_{k} = P\left( {1_{k} \left( y \right) = 1} \right) = P\left( {l_{k} \le \left. {\tau_{k} } \right|u_{k} } \right),$$

and the $n - 1$ probabilities $\pi_{k}$ should satisfy the relation $\sum\nolimits_{k = 1}^{n - 1} {\pi_{k} < 1.}$ This constraint must be added to the multivariate binary threshold model.

As for ITM, there are $\left( {n + 2} \right)\left( {n - 1} \right)/2$ parameters to estimate i.e. $n - 1$ thresholds and $n\left( {n - 1} \right)/2$ variance covariance parameters.

The relationship between the thresholds of MTM and ITM is as follows:

$$\tau_{ik}^{\text{MTM}} = {{\Phi }}^{ - 1} \left( {{{\Phi }}\left( {\tau_{ik}^{\text{ITM}} } \right) - {{\Phi }}\left( {\tau_{{i\left( {k - 1} \right)}}^{\text{ITM}} } \right)} \right).$$

Under the latter models, one cannot really consider that the expression of the ordinal trait is controlled by two sets of genes. The combination of elementary genetic values for each individual results in the heteroscedasticity of the global trait. In this model, the heritability can be calculated for each class as:

$$h_{k}^{2} = \frac{{\sigma_{{u_{k} }}^{2} }}{{\sigma_{{u_{k} }}^{2} + 1}}.$$

The different parameterizations of the models are summarized in Table 1. HTM’ and ITM can be fitted using the ASReml software.

**Table 1:** Main characteristics of the different models

Models	Number of parameters	Thresholds	Underlying
		Population	
*Homoscedastic*			
TM	$n$	${\mathbf{t}}^{*} = \left\{ {{\mathbf{t}}_{1}^{*} ,{\mathbf{t}}_{2}^{*} , {\mathbf{t}}_{3}^{*} , \ldots ,{\mathbf{t}}_{n - 1}^{*} } \right\}$	${\text{Y }}\sim{\text{N}}\left( {0, 1} \right)$
*Heteroscedastic*			
HTM	$n + 2$	${\mathbf{t}} = \left\{ {0, 1, {\mathbf{t}}_{3} , \ldots , {\mathbf{t}}_{n - 1} } \right\}$	${\text{Y }}\sim{\text{N}}\left( {\mu + u,f^{2} (\eta ,{\mathbf{v}}} )\right)$
HTM’	$n + 2$	${\mathbf{t^{\prime}}} = \left\{ {{\mathbf{t}}_{1}^{'} ,{\mathbf{t}}_{2}^{'} , {\mathbf{t}}_{3}^{'} , \ldots ,{\mathbf{t}}_{n - 1}^{'} } \right\}$	${\text{Y }}\sim{\text{N}}\left( {0, 1} \right)$
ITM	$\left( {n + 2} \right)\left( {n - 1} \right)/2$	${\mathbf{t}}^{{{\prime \prime }}} = \left\{ {{\mathbf{t}}_{1}^{{{\prime \prime }}} ,{\mathbf{t}}_{2}^{{{\prime \prime }}} , {\mathbf{t}}_{3}^{{{\prime \prime }}} , \ldots ,{\mathbf{t}}_{n - 1}^{{{\prime \prime }}} } \right\}$	${\text{Y }}\sim{\text{N}}\left( {0, 1} \right)$
		Animal *i*	
*Homoscedastic*			
TM	$n$	${\text{t}}_{i}^{*} = \left\{ {{\text{t}}_{1i}^{*} , {\text{t}}_{2i}^{*} , {\text{t}}_{3i}^{*} , \ldots , {\text{t}}_{{\left( {n - 1} \right)i}}^{*} } \right\}$ ${\text{t}}_{.i}^{*} = {\text{t}}_{i} + \mu_{i} + u_{i}$	${\text{y}}_{i} = {{\upvarepsilon }}_{i}$
*Heteroscedastic*			
HTM	$n + 2$	${\text{t}}_{i} = \left\{ {0, 1 {\text{t}}_{3i} , \ldots , {\text{t}}_{{\left( {n - 1} \right)i}} } \right\}$	${\text{y}}_{i} = {{\mu }}_{i} + {u_{i}} + {{\upvarepsilon }}_{i}$
HTM’	$n + 2$	${\text{t}}_{i}^{'} = \left\{ {{\text{t}}_{1i}^{'} , {\text{t}}_{2i}^{'} , {\text{t}}_{3i}^{'} , \ldots , {\text{t}}_{{\left( {n - 1} \right)i}}^{'} } \right\}$ ${\text{t}}_{.i}^{'} = {\text{t}}_{i}^{'} + \mu_{i}^{'} + u_{i}^{'} + {\text{v}}_{i}^{'}$	${\text{y}}_{\text{i}} = {{\upvarepsilon }}_{i}$
ITM	$\left( {n + 2} \right)\left( {n - 1} \right)/2$	${\text{t}}_{i}^{{{\prime \prime }}} = \left\{ {{\text{t}}_{1i}^{{{\prime \prime }}} , {\text{t}}_{2i}^{{{\prime \prime }}} , {\text{t}}_{3i}^{{{\prime \prime }}} , \ldots , {\text{t}}_{{\left( {n - 1} \right)i}}^{{{\prime \prime }}} } \right\}$ ${\text{t}}_{.i}^{{{\prime \prime }}} = {\text{t}}_{i}^{{{\prime \prime }}} + \mu_{i}^{{{\prime \prime }}} + u_{i}^{{{\prime \prime }}}$	${\text{y}}_{i} = {{\upvarepsilon }}_{i}$

### Numerical application

#### Data

These models were applied to study litter size in Romane sheep. All data (litter size, environmental variation factors and pedigrees over ten generations) were extracted from the national database for genetic evaluation and research managed by the Institut de l’Elevage (French Livestock Institute) and the Centre de Traitement de l’Information Génétique (Genetic Information Processing Center, Jouy-en-Josas, France). Data consisted of records on litter size of 11,073 multiparous ewes from 23 flocks with at least four lambings from 2000 to 2013. The mean prolificacy (2.13) in this sample was high and consequently there was a high global variability in litter size. The main features of this dataset are in Table 2. To avoid extreme case problems [14], litter sizes of more than four lambs were pooled into a single class for all analyses, which resulted in four classes of litter size i.e. [1, 2, 3, 4+].

**Table 2:** Features of Romane sheep

*Item*	
Number of records	19,671
Number of animals	11,073
Number of sires	1096
Number of dams	6550
Mean	2.13
*Distribution of litter size (%)*	
Single	17.70
Twin	55.01
Triplet	24.18
Quadruplet and +	3.11

The fixed effects included in the analysis were selected to be as similar as possible to those found in others studies [15, 16]. This was done by comparing the likelihood-ratio of all the possible linear mixed models which included the following fixed effects: age of ewe (nine levels), time interval between lambing in months (seven levels), parity (seven levels), age of the ewe at first lambing in months (nine levels), season of lambing (two levels), as well as the random effects: flock and ewe effect. For this step of the analysis, the ewe effect was considered as random but the genetic relationships between individuals were ignored.

#### Estimation of parameters and comparison of methods

The three threshold models TM, HTM’ and ITM described above were applied to this data. The classical TM, used as a basis for comparison, included the previously selected fixed effects and the ewe as both a random genetic effect ($u^{*}$) and a random permanent environmental effect $\left( {p^{*} } \right)$ (i.e. ${\mathbf{t}}_{i}^{*} = \varvec{x}_{i} {\varvec{\beta}} + u_{i}^{*} \mathbf1^{{\mathbf{T}}} + p_{i}^{*} \mathbf1^{{\mathbf{T}}}$). The homothetic threshold model HTM’ considered that fixed, genetic and permanent effects were common for all thresholds and threshold-specific random genetic effects with constrained genetic (co)variance to ensure homothetic translation (i.e. ${\mathbf{t}}_{i}^{'} = \varvec{x}_{i} {\varvec{\beta}} + u_{i}^{'} \mathbf1^{{\mathbf{T}}} + p_{i}^{'} \mathbf1^{{\mathbf{T}}} + {\mathbf{v}}_{\varvec{i}}^{\varvec{'}}$). Finally, an additional extension resulting in the independent threshold model ITM was fitted and it considered that the fixed and permanent effects were common for all thresholds and included a genetic effect that was specific to each threshold with no constrained genetic (co)variance (i.e. ${\mathbf{t}}_{i}^{{{\prime \prime }}} = \varvec{x}_{i} {\varvec{\beta}} + p_{i}^{{{\prime \prime }}} \mathbf1^{{\mathbf{T}}} + {\mathbf{v}}_{\varvec{i}}^{{{\prime \prime }}}$).

ASReml software [17] was used to implement all the models.

Models were compared using the likelihood ratio test for nested models (TM vs. HTM’, LRT df = 2; TM vs. ITM, LRT df = 5) and the Bayesian Information Criterion (BIC) for non-nested models (${\text{BIC}} = { \ln }\left( {N - p} \right)^{*} c - 2{ \ln }L$, HTM’ vs. ITM, where $L$ is the restricted maximum likelihood of the model, $N$ the number of observations, $p$ and $c$ the number of fixed effects and covariance parameters) [18]. The models were also compared based on their ability to predict new records as follows: 75 % of records were used to estimate parameters that were used to predict the remaining 25 %. Five replicates of this design were sampled at random. To evaluate the predictive ability of the models, MSEP (mean square error of prediction) statistic was computed as:

$${\text{MSEP}} = \frac{1}{\text{M}}\mathop \sum \limits_{j = 1}^{\text{M}} \left( {\frac{1}{m}\mathop \sum \limits_{i = 1}^{m} \left( {y_{ij} - \hat{y}_{ij} } \right)^{2} } \right),$$

where $y$ and $\hat{y}$ correspond to the observed and predicted litter size, $m$ is the number of data in the validation subset and ${\text{M}}$ is the subset number.

For ewes with observations, we compared the raw means and variances of their litter sizes to the corresponding predicted values on the observable scale obtained with the different models. The raw mean and variance were calculated for each individual from the observed data. The predicted values were calculated as:

$$\hat{y}_{i} = \mathop \sum \limits_{k = 1}^{4} k\hat{\pi }_{i,k} ,$$

and

$$\hat{\sigma }_{i}^{2} = \mathop \sum \limits_{k = 1}^{4} k^{2} \hat{\pi }_{i,k} - \left( {\mathop \sum \limits_{k = 1}^{4} k\hat{\pi }_{i,k} } \right),$$

where $\hat{\pi }_{i,k}$ was the probability that the litter size of a ewe $i$ was $k$. These probabilities were calculated using $\hat{\pi }_{i,k} = {{\Phi }}\left( {\hat{\tau }_{i,k} } \right) - {{\Phi }}\left( {\hat{\tau }_{i,k - 1} } \right)$ with as above $\tau_{0} = - \infty$ and $\tau_{4} = + \infty$ ($\hat{\tau }_{i,k}$ corresponds to $\hat{\tau }_{i,k}^{*} , \hat{\tau }_{i,k}^{'}$ or $\hat{\tau }_{i,k}^{{{\prime \prime }}}$ depending on the model). To measure the ability of the models to break the link between $\overline{{\hat{y}}}_{i} = t$ and $\hat{\sigma }_{i}^{2} = z,$ we used the following criterion:

$${\text{B}} = \frac{{E\left( {Var\left( {\left. z \right|t} \right)} \right)}}{Var\left( z \right)},$$

which ranges from 0 to 1. The criterion ${\text{B}}$ was 0 if $E\left( {Var\left( {\left. z \right|t} \right)} \right) = 0,$ which means that a single value of the mean has a single value of the variance. The criterion ${\text{B}}$ was 1 if $E\left( {Var\left( {\left. z \right|t} \right)} \right) = 1,$ which means that a single value of the mean has different variance values. Because the density associated to the variance $Var\left( {\left. z \right|t} \right)$ is unknown, data were ordered in ascending order of litter size and were discretized into $s$ subgroups, and the variance of *y* in each subgroup $\hat{\sigma }_{m}^{2}$ was calculated in order to calculate:

$$E\left( {Var\left( {\left. z \right|t} \right)} \right) = {\text{E}}\left( {\left. {\hat{\sigma }_{{{\text{y}}_{\text{i}} }}^{2} } \right|\overline{{\hat{y}}}_{\text{i}} } \right) = \mathop \sum \limits_{m = 1}^{s} \hat{\sigma }_{m}^{2} /s.$$

The subgroup number was defined in order to get a sufficient amount of data in each subgroup to obtain a good estimation of the subgroup variance. The intensity of the dispersion of each model was also assessed by calculating the correlation, either for the mean or the variance, between the observed values and the predicted values on the observable scale.

## Results

### Comparison of models

The values of the criteria (LRT, BIC, MSE and MSEP) used to compare the adjustment quality of the models are in Tables 3 and 4.

**Table 3:** Values obtained from likelihood-ratio, BIC tests, B criteria, and mean squared error (MSE) between observed and fitted values in the different models

Models/tests	Likelihood-ratio (LRT)	BIC test	B	MSE of mean	MSE of variance
TM	TM versus HTM': ≫11 (Reject H0 *P* value <0.001) TM versus ITM: ≫16 (Reject H0 *P* value <0.001)	54,005	0.013	0.07	0.13
HTM’		49,517	0.51	0.07	0.10
ITM		50,663	0.42	0.07	0.10

*TM* threshold model, *HTM’* homothetic threshold model, *ITM* independent threshold model, *B* B criteria

**Table 4:** Values obtained from mean squared error of prediction (MSEP) and B criteria on the validation subset in the different models

Models/tests	MSEP	B criteria
Threshold model (TM)	0.473	0.005
Homothetic threshold model (HTM’)	0.471	0.363
Independent threshold model (ITM)	0.481	0.371

The results showed that the homoscedastic model should be rejected and that the models that account for genetic heterogeneity of litter size distribution should be favored. Among the heteroscedastic models, BIC values were lower with HTM’ than with ITM. MSE estimates of the mean were similar for the three models, whereas MSE estimates of the variance differed and were higher with TM than with HTM’ (0.13 with TM vs. 0.10 with HTM’). The differences between MSEP values are small, which is explained by the fact that this parameter can estimate prediction error for the level of the trait only and not for its variability. To evaluate the ability of the models to predict variance, we calculated the B criteria on the validation subset by discretizing the whole data into 200 adjacent subgroups. The results are in Table 4. Based on the B criteria, HTM’ and ITM were more efficient than TM (0.36 for HTM’, 0.37 for ITM vs. 0.005 for TM).

### Homoscedastic threshold model used as reference (TM)

**Fig. 1:**
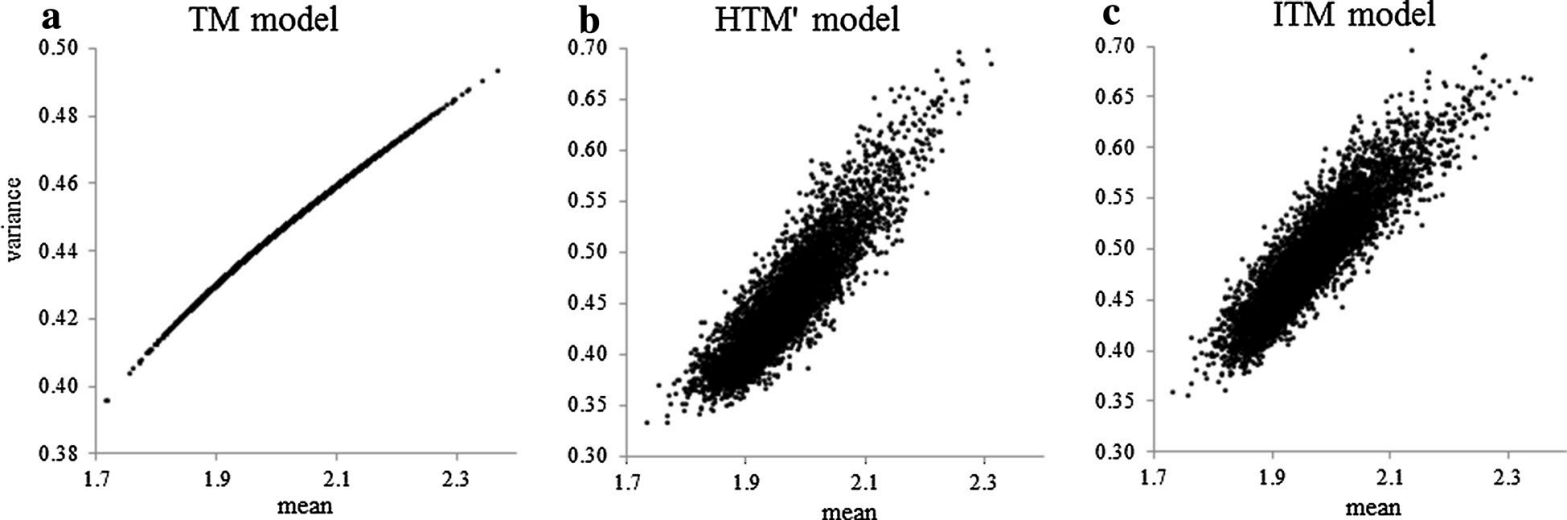
Scatterplots of estimated mean vs. estimated variance across the observed range for the three models. **a** TM model. **b** HTM' model. **c** ITM model

With TM, the genetic additive variance of the liability $\sigma_{{u^{*} }}^{2}$ was equal to $0.079$ and the variance of the permanent environmental $\sigma_{{p^{*} }}^{2}$ effect was equal to $0.034$, which resulted in a heritability $h^{2}$ of 0.07. The correlation between the predicted and observed means was very high ($r = 0.80$) (Table 5), but it was low between the predicted and observed variances ($r = 0.20$). In spite of the curvilinearity between the predicted means and variances, the correlation between these variables was high ($r = 0.99$) and close to 1, and the ${\text{B}}$ parameter was equal to 0.013 (Table 3), which reveals the one-to-one relationship between these variables when this homoscedastic threshold model was used as shown on the scatterplot of Fig. 1a.

**Table 5:** Correlations between the predicted and observed means as well as the predicted observed variance with the different models

Model	Predicted mean versus observed mean	Predicted variance versus observed variance
TM	0.80	0.21
HTM’	0.79	0.61
ITM	0.78	0.59

*TM* threshold model, *HTM’* homothetic threshold model, *ITM* independent threshold model

### Homothetic threshold model (HTM’)

The genetic additive variances obtained with HTM’ are in Table 6. By design, the correlation between the genetic values associated to thresholds (${\text{v}}$) was equal to 1 in this model and the variance $\sigma_{{\nu^{\prime}.1}}^{2}$ was equal to 0. Moreover, and as expected due to the homothetic relationship, we observed an increase of $\sigma_{{\nu^{\prime}.3}}^{2}$ compared to $\sigma_{{\nu^{\prime}.2}}^{2}$. The variances were equal to 0.04, 0.05 and 0.20 for $\sigma_{{u^{\prime}.1}}^{2} , \sigma_{{\nu^{\prime}.2}}^{2} ,{\text{and }} \sigma_{{\nu^{\prime}.3}}^{2}$, respectively. The variance of the permanent environmental effect was equal to 0.015.

**Table 6:** Genetic variance and correlation between thresholds for Romane sheep using the HTM’ model

Thresholds/genetic variance	$\sigma_{{u^{\prime}.1}}^{2}$	$\sigma_{{\nu^{{\prime }} .2}}^{2}$	$\sigma_{{\nu^{{\prime }} .3}}^{2}$
	0.04	0.13	0.13
		0.05	1
			0.20

By transforming the results of the HTM’ parameterization to those of HTM, we could estimate the variance of the genetic effects that affect the mean of the liability as well as the correlation between these effects. The values found for the mean of the liability, the scaling function and the correlation between effects were $\sigma_{u}^{2} = 0.069$, $\sigma_{\nu }^{2} = 0.020$ and $\rho = - 0.40$, respectively. The variance of the permanent effect was $\sigma_{p}^{2} = 0.015$ whereas, by design, the mean of the residual variance was $\eta^{2} = 1$. Since the model fitted for the environmental variance did not include fixed effects but only a general mean and a genetic effect, the heritability of the liability mean could be estimated independently of a given environment and was as follows:

$$h_{l}^{2} = \frac{{\sigma_{u}^{2} }}{{\sigma_{u}^{2} + \sigma_{p}^{2} + \eta^{2} + \sigma_{\nu }^{2} }} = 0.062.$$

The correlation between observed and predicted means was high ($r = 0.79$) and not different from the value obtained with TM. This contrasts with the correlation between predicted and observed variances ($r = 0.61$). Although the prediction of the variances was still lower than that of the means, it was much higher than with TM. HTM’ could therefore break the link between the predicted means and variances and the B parameter was equal to 0.51 (Table 3) even if the correlation between these variables was still high ($r = 0.88$). The scatterplot in Fig. 1b shows this variability of the predicted variance for a given predicted mean.

In this homothetic translation model, the gap ${{\Delta }}_{1 - 2}$ between the two first thresholds is an indirect measure of its variability for each individual. As an example, we estimated the distribution of the litter size of two individuals having the same average litter size but different variances (Table 7). For these individuals, the values of the mean and variance estimated with HTM’ are close to the observed values of the mean and variance. The ratio is constant for the two individuals, and the difference in percentage of grand-offspring born as quadruplets or more can be large (0 vs. 6 %).

**Table 7:** Observed values (prolificacy, variance) and estimated values with HTM’ [thresholds, different gap between the first thresholds (${{\Delta }}_{1 - 2}$), ratio (R), prolificacy, variance, and percentage of the simple (% LS 1), double (% LS 2), triplet (% LS 3) and quadruplet (% LS 4)] for two individuals

Observed values			Estimated values with HTM’ model										
Ind	Prolificacy	Variance	$\tau_{1}^{'}$	$\tau_{2}^{'}$	$\tau_{3}^{'}$	${{\Delta }}_{1 - 2}$	$R$	Prolificacy	Variance	% LS1	% LS2	% LS3	% LS4+
1	2.00	0.50	−0.63	0.92	2.16	1.55	1.8	2.09	0.54	16	53	25	6
2	2.00	0.33	−0.89	0.86	2.28	1.75	1.8	2.02	0.38	21	64	15	0

### Independent threshold model (ITM)

The genetic variances and correlations obtained with ITM are in Table 8. Genetic variances were small, but larger for the second and third thresholds than for the first threshold. Genetic correlations were moderate ($\rho > 0.4$) and it was highest between $\nu_{2}^{{{\prime \prime }}}$ and $\nu_{3}^{{{\prime \prime }}}$. Using the transformation formulas described in “Appendix 2”, it was possible to estimate the genetic variance associated with twin lambing ($\sigma_{ls = 2}^{2} = 0.14$), the corresponding heritability ($h_{ls = 2}^{2} = 0.128$) as well as the genetic correlation between the values for the first and the second classes of litter size ($\rho = 0.44$). The correlations between the predicted and observed means ($r = 0.78$) and between the predicted and observed variances ($r = 0.59$) were close to those obtained with HTM (Table 8). The ${\text{B}}$ parameter (${\text{B}} = 0.42$, Table 3), the correlation between the predicted means and variances ($r = 0.91$) and the scatter plot (Fig. 1c) were also similar to those obtained with HTM’. The Spearman rank correlations between the thresholds of HTM’ and ITM were high (Table 9).

**Table 8:** Genetic variance and correlation between thresholds for Romane sheep using the ITM model

Thresholds/genetic variance	$\sigma_{{\nu^{\prime\prime}.1}}^{2}$	$\sigma_{{\nu^{\prime\prime}.2}}^{2}$	$\sigma_{{\nu^{\prime\prime}.3}}^{2}$
	0.035	0.631	0.430
		0.138	0.921
			0.176

**Table 9:** Spearman rank correlations between the HTM’ and ITM models

First threshold	0.988
Second threshold	0.989
Third threshold	0.988

## Discussion

A genetic heteroscedastic model was previously proposed [6] to analyze categorical variables and break down the link between mean and variance that holds with the classical homoscedastic threshold model. This model originated from the combination of two base models: the homoscedastic threshold model for categorical data [19] and the structural heteroscedastic model for continuous variables [2]. In this paper, we studied further extensions of this model by analyzing the relationship that exists between categories and in particular by releasing the main constraint. 

In this paper, we propose two kinds of heteroscedastic models, both with different parameterizations. The first type (HTM/HTM’) was fully derived from the canalization model proposed for continuous traits [2] with which it was easy to draw comparisons. The second type consisted in relaxing the relationship that exists between thresholds (translation in TM or homothetic translation in HTM) and led to a model that considers each threshold independently i.e. ITM. We demonstrated that this model was equivalent to a constrained multiple-trait model MTM on each class of the ordinal trait. This second type of model is much more flexible than HTM and its application in selection would be of particular interest because it allows for each class to be controlled through a multiple selection index [20] with specific environmental effects and economic weights for each class. Modeling each class of the trait to control its distribution or modeling only the most important class are trivial issues and have already been considered [21], however the correct multiple trait model must include a constraint on the sum of the probabilities of the $n - 1$ binary variables. In spite of their potential interest, the use of this kind of model (ITM or MTM) for genetic evaluation and selection would be limited since they require a larger number of parameters to be estimated than HTM or HTM’ and would probably show convergence problems when the number of classes exceeds three. Furthermore, their biological interpretation is difficult; for instance we could not link the parameters of ITM or MTM to the biological model of Schneider and Lyman [22], which assumes the existence of different genes that control the mean and the variance of a trait.

We compared TM, HTM’ and ITM through the analysis of data on litter size from both Romane and Rouge de l’Ouest sheep (the latter results are not shown). The low heritability of the liability to litter size obtained with an homoscedastic threshold model (i.e. TM) agreed with previous studies that used TM [23–25]. However, LRT, BIC, and MSE parameters showed that this homoscedastic model was less successful than both heteroscedastic models. Consequently, the distribution of sheep litter size shows heteroscedasticity of genetic origin as revealed by both the homothetic HTM’ and independent threshold ITM models.

Taking the genetic heteroscedasticity in HTM’ and the subsequent transformation of the parameters to those of HTM into account did not substantially change the heritability of the liability to litter size compared to TM. However, with HTM, the additive genetic variance of the variability of the trait was smaller than the corresponding variance of its mean. This is in line with results in the literature that showed that when additive genetic variances of the mean and the variability of a trait were jointly estimated, their values were smaller for the variability than for the mean [6, 9, 26–31]. This seems to be a common rule regardless of the methodology used for such estimations, the trait or the species. In contrast, the genetic correlation between the mean of a trait and its variability is highly variable and covers the whole range of its variation interval. For instance, in a mouse population, genetic correlations of −0.93 and 0.97 were reported for litter size and individual birth weight, respectively [29]. In our study, we found a negative genetic correlation between the mean and the variability of sheep litter size with HTM’ ($\rho = - 0.40$). As highlighted by Yang et al. [32], this parameter might be affected by an artefact due to the scale of measurement or skewness of the data, although with the threshold model no such skewness affected this correlation. However, a study on another French sheep breed (Rouge de l’Ouest) for which the mean prolificacy (prolificacy = 1.80; LS3+ = 12.5 %) is lower than in Romane sheep (results not shown) reported a similar value i.e. −0.25.

Relaxing the homothetic constraint between the thresholds of HTM’ did not improve the fit of the model or the predictive ability of the data as might be expected, but instead increased the BIC values due to the larger number of parameters to estimate in ITM. Moreover, although the constraint on the genetic correlation between thresholds was relaxed in ITM, the correlation between the second and third thresholds remained close to 1 and the correlation between the first and second thresholds was high; in addition, the Spearman rank correlations between the thresholds of HTM’ and ITM were close to 1 (Table 9). This suggests that the true nature of the link between the thresholds is almost a homothetic translation in sheep and that the distributions of litter size classes are not independent but linked by a strong common law. It also means that the concept of sensitivity thresholds, which transform individual Gaussian variables that differ in mean and variance into the observed categories, might be relevant.

Nonetheless in our study, only genetic effects were considered as threshold-specific in ITM in order to compare the different models using LRT or BIC criteria. Of course, threshold-specific fixed effects and permanent effects could be included in ITM. In the same way, threshold-specific fixed effects and permanent effects could be included in HTM’. Unfortunately, HTM with a threshold-specific permanent effect did not converge and threshold-specific fixed factors with proportional effects cannot be implemented in ASReml. It may be possible to estimate fixed effects in the residual variance using an iterative process that tests the influence of each effect on the variance of the model. We did not test such algorithms on our data and it would probably be a slow and difficult process. Another solution would be to modify existing software for canalizing selection of a continuous trait in order to take into account the discrete characteristics of the data.

**Fig. 2:**
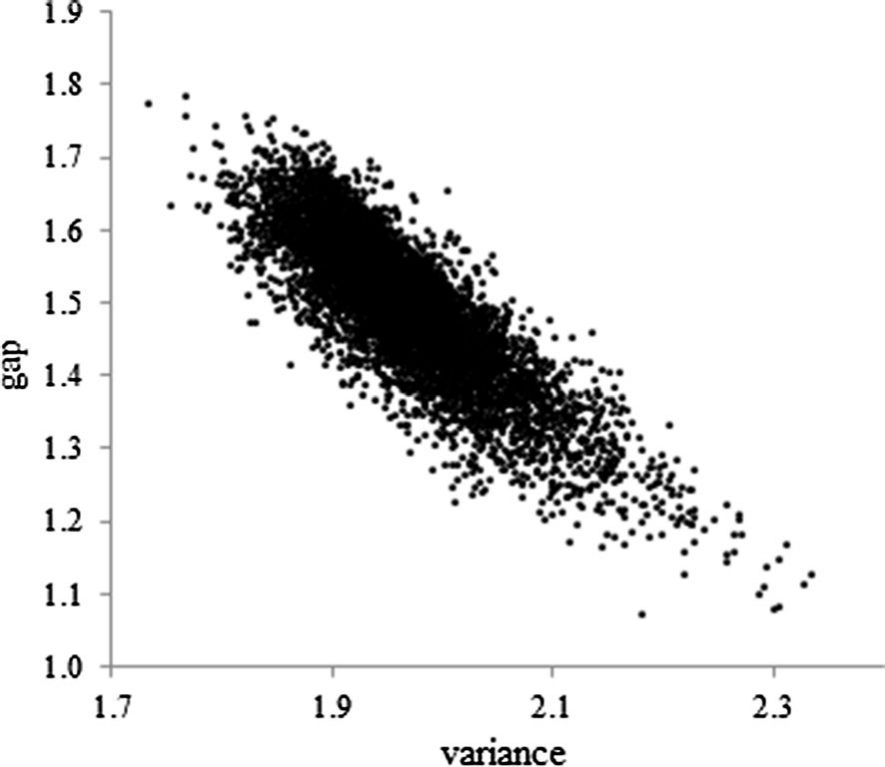
Scatterplot of estimates of the gap between the threshold and the mean predicted by the HTM model

Using ITM, Martin et al. [33] showed that the polymorphism of a single gene affected not only the mean prolificacy in Lacaune sheep, but also modified its variability by changing the gap between the thresholds in a homothetic way. A superiority of the homothetic model (HTM’), as well as a similar genetic variability of the liability to litter size were found for Rouge de l’Ouest sheep (results not shown), thus covering the whole range of prolificacy in breeds for which canalization might be of interest. By design, the gap between the first two thresholds estimated for each individual in HTM’ is inversely proportional to the individual residual variance of HTM (Fig. 2). This criterion has been used to compare the variability of about 50 French sheep breeds [34].

## Conclusions

This study shows that we can model the variabilities (mean and variance) of a discrete trait using a suitable model, namely the HTM’ model. Thus, the canalization of discrete traits is possible and the gap between the first two thresholds estimated for each individual in this model could be used to estimate the breeding value for the variability of litter size independently from that of the mean.

## Appendix 1

In this section we describe how we calculated the parameters $\sigma_{u}^{2}$, $\sigma_{\nu }^{2}$ and $\rho_{u\nu }$ of a HTM model based on the HTM’ estimates. Let $t_{k}^{'}$ be the *k*^t^^h^ threshold of the HTM’ model with ${\mathbf{t}}_{.i}^{'} = \mu^{'} + u_{i}^{'} \mathbf1^{{\mathbf{T}}} + {\mathbf{v}}_{\varvec{i}}^{'}$, a simple variable transformation gives:

$$\left\{ {\begin{array}{l} {t_{2}^{'} = \frac{{t_{2} - \left( {\mu + u} \right)}}{{f\left( {\eta + \nu } \right)}}} \\ {t_{1}^{'} = \frac{{t_{1} - \left( {\mu + u} \right)}}{{f\left( {\eta + \nu } \right)}}} \\ \end{array} } \right..$$

Given that $t_{1} = 0$ and $t_{2} = 1$ in HTM and $t_{1.}^{'} = \mu_{1}^{'} + u^{'}$ and $t_{2.}^{'} = \mu_{2}^{'} + u^{'} + \nu_{2}^{'}$ in HTM’, we obtain:

$$\left\{ {\begin{array}{l} {f\left( {\eta ,\nu } \right) = \frac{1}{{\mu_{2}^{'} - \mu_{1}^{'} + \nu_{2}^{'} }}} \\ \quad { = \frac{1}{{\mu_{2}^{'} - \mu_{1}^{'} }} \left( {\frac{1}{{1 + \nu_{2}^{'} /\mu_{2}^{'} - \mu_{1}^{'} }}} \right)} \\ {u = - \mu - f\left( {\eta ,\nu } \right)\left( {\mu_{1}^{'} + u^{'}} \right)}. \\ \end{array} } \right.$$

Considering that the ratio $\nu_{2}^{'} /\mu_{2}^{'} - \mu_{1}^{'}$ is small in comparison to 1, we applied Taylor’s expansion to the previous equation:

$$\Rightarrow \left\{ {\begin{array}{l} {f\left( {\eta , \nu } \right) = \frac{1}{{\mu_{2}^{'} - \mu_{1}^{'} }} - \frac{{\nu_{2}^{'} }}{{\left( {\mu_{2}^{'} - {{\upmu }}_{1}^{'} } \right)^{2} }}} \\ {} \\ {u = - \mu - \frac{1}{{\mu_{2}^{'} - \mu_{1}^{'} }}\left( {1 - \frac{{\nu_{2}^{'} }}{{\mu_{2}^{'} - \mu_{1}^{'} }}} \right)\left( {\mu_{1}^{'} + u^{'}} \right)} \\ \end{array} } \right.$$

Thus, In the case of a linear function $f$ and given that $\nu_{2}^{{\prime }} = \left( {\mu_{2}^{{\prime }} - \mu_{1}^{{\prime }} } \right)\nu^{{\prime }} ,$ we obtain:

$$\Rightarrow \left\{ {\begin{array}{l} {\nu = C_{1} - \frac{{\nu^{{\prime }} }}{{\mu_{2}^{{\prime }} - \mu_{1}^{{\prime }} }}} \\ \\ {u = C_{2} + \nu^{{\prime }} \left( {\frac{{\mu_{1}^{{\prime }} }}{{\mu_{2}^{{\prime }} - \mu_{1}^{{\prime }} }}} \right) - u^{{\prime }} \left( {\frac{1}{{\mu_{2}^{{\prime }} - \mu_{1}^{{\prime }} }}} \right) + u^{{\prime }} \nu^{{\prime }} \left( {\frac{1}{{\mu_{2}^{{\prime }} - \mu_{1}^{{\prime }} }}} \right)} \\ \end{array} } \right.,$$

where $C_{1} = \frac{1}{{\mu_{2}^{'} - \mu_{1}^{'} }} - \eta$ and $C_{2} = - \mu - \frac{{\mu_{1}^{'} }}{{\mu_{2}^{'} - \mu_{1}^{'} }}$ are constant terms.

Therefore, given that the variance of the product of two centered random variables $X$ and $Y$ is $\sigma_{X*Y}^{2} = \sigma_{X}^{2} \sigma_{Y}^{2} + \left( {\sigma_{X,Y} } \right)^{2}$ and that $\sigma_{XY,Y} = 0$, we obtain:

$$\left\{ {\begin{array}{l} {\sigma_{\nu }^{2} = \frac{{\sigma_{{\nu^{\prime}}}^{2} }}{{\left( {\mu_{2}^{'} - \mu_{1}^{'} } \right)^{2} }}} \\ \\ {\sigma_{u}^{2} = \frac{1}{{\left( {\mu_{2}^{'} - \mu_{1}^{'} } \right)^{2} }}\left( {\left( {\mu_{1}^{'} } \right)^{2} \sigma_{{\nu^{\prime}}}^{2} + \sigma_{{u^{\prime}}}^{2} + \sigma_{{\nu^{\prime}}}^{2} \sigma_{{u^{\prime}}}^{2} + \left( {\sigma_{{\nu^{\prime},u^{\prime}}} } \right)^{2} - 2\mu_{1}^{'} \sigma_{{\nu^{\prime},u^{\prime}}} } \right)} \\ \end{array}} \right..$$

The correlation between $\nu$ and $u$ is: $\rho_{u\nu } = \frac{{\sigma_{u\nu } }}{{\sigma_{u} \sigma_{\nu } }},$ with:

$$\begin{aligned} \sigma_{\nu u} & = {\text{cov}}\left( { - \frac{{{{\upnu^{\prime}}}}}{{\mu_{2}^{'} - \mu_{1}^{'} }},C_{2} + \nu^{\prime}\left( {\frac{{\mu_{1}^{'} }}{{\mu_{2}^{'} - \mu_{1}^{'} }}} \right) - u^{\prime}\left( {\frac{1}{{\mu_{2}^{'} - \mu_{1}^{'} }}} \right) + u^{\prime}\nu^{\prime}\left( {\frac{1}{{\mu_{2}^{'} - \mu_{1}^{'} }}} \right)} \right) \\ & = \frac{1}{{\left( {\mu_{2}^{'} - \mu_{1}^{'} } \right)^{2} }}\left( {\sigma_{{\nu^{\prime},u^{\prime}}} - \mu_{1}^{'} \sigma_{{\nu^{\prime}}}^{2} } \right). \\ \end{aligned}$$

## Appendix 2

In this section, we describe how we calculated the genetic variance ($u_{2}^{{{\prime \prime }*}}$) in the MTM model based on the genetic variances $\left( {u_{1}^{{{\prime \prime }}} ,u_{2}^{{{\prime \prime }}} } \right)$ of the ITM model.

The probability that the trait be of class 1 (2, or 3, etc.) is the same for both models, thus:

$$\left\{ {\begin{array}{l} {P\left( {y = 1} \right)_{ITM} = P\left( {y = 1} \right)_{MTM} } \\ \\ {P\left( {y = 2} \right)_{ITM} = P\left( {y = 2} \right)_{MTM} } \\ \end{array} } \right.,$$

$$\Rightarrow \left\{ {\begin{array}{l} {L\left( {t_{1}^{{{\prime \prime }}} } \right) = L\left( {t_{1}^{{{\prime \prime }*}} } \right)} \\ {L\left( {t_{2}^{{{\prime \prime }}} } \right) - L\left( {t_{1}^{{{\prime \prime }}} } \right) = L\left( {t_{2}^{{{\prime \prime }*}} } \right)} \\ \end{array} } \right.,$$

$$\Rightarrow \left\{ {\begin{array}{l} {\frac{1}{{1 + e^{{ - t_{1}^{{{\prime \prime }}} }} }} = \frac{1}{{1 + e^{{ - t_{1}^{{{\prime \prime }*}} }} }}} \\ {\frac{1}{{1 + e^{{ - t_{2}^{{{\prime \prime }}} }} }} - \frac{1}{{1 + e^{{ - t_{1}^{{{\prime \prime }}} }} }} = \frac{1}{{1 + e^{{ - t_{2}^{{{\prime \prime }*}} }} }}} \\ \end{array} } \right.,$$

where $L$ is the Logit distribution function.

$$\Rightarrow \left\{ {\begin{array}{l} {{\text{e}}^{{ - {\text{t}}_{ 1}^{{{\prime \prime } *}} }} = {\text{e}}^{{ - {\text{t}}_{ 1}^{{{\prime \prime } *}} }} } \\ \\ {{\text{e}}^{{ - {\text{t}}_{ 2}^{{{\prime \prime } *}} }} = \frac{ 1}{{\frac{{ 1 +{\text{ 2e}}^{{ - {\text{t}}_{ 2}^{{{\prime \prime }}} }} +{\text{ e}}^{{ - \left( {{\text{t}}_{ 2}^{{{\prime \prime }}} +{\text{ t}}_{ 1}^{{{\prime \prime }}} } \right)}} }}{{{\text{e}}^{{ - {\text{t}}_{ 2}^{{{\prime \prime }}} }} - {\text{e}}^{{ - {\text{t}}_{ 1}^{{{\prime \prime }}} }} }}}}} \\ \end{array} } \right.$$

$$\Rightarrow \left\{ {\begin{array}{l} {t_{1}^{{{\prime \prime }*}} = t_{1}^{{{\prime \prime }}} } \\ \\ {t_{2}^{{{\prime \prime }*}} = {\text{ln}}\left( {\frac{{1 + 2e^{{ - t_{2}^{{{\prime \prime }}} }} + e^{{ - \left( {t_{2}^{{{\prime \prime }}} + t_{1}^{{{\prime \prime }}} } \right)}} }}{{e^{{ - t_{2}^{{{\prime \prime }}} }} - e^{{ - t_{1}^{{{\prime \prime }}} }} }}} \right)} \\ \end{array} } \right.$$

Thus, the variance $\sigma_{{u_{2}^{{{\prime \prime }*}} }}^{2}$ is:

$$\sigma_{{u_{2}^{{{\prime \prime }*}} }}^{2} = \sigma_{{{ \ln }\left( {\frac{{1 + 2e^{{ - t_{2}^{{{\prime \prime }}} }} + e^{{ - \left( {t_{2}^{{{\prime \prime }}} + t_{1}^{{{\prime \prime }}} } \right)}} }}{{e^{{ - t_{2}^{{{\prime \prime }}} }} - e^{{ - t_{1}^{{{\prime \prime }}} }} }}} \right)}}^{2} .$$

We see that, from the parameters of the ITM model, we can deduce the parameters of the MTM model.

## References

[1] Scheiner SM, Lyman RF (1989). The genetics of phenotypic plasticity I. Heritability. J Evol Biol.

[2] SanCristobal-Gaudy M, Elsen JM, Bodin L, Chevalet C (1998). Prediction of the response to a selection for canalisation of a continuous trait in animal breeding. Genet Sel Evol.

[3] Garcia M, David I, Garreau H, Ibañez-Escriche N, Mallard J, Masson JP, et al. Comparisons of three models for canalising selection or genetic robustness. In: Proceedings of the 60th annual meeting of the European association for animal production: 24–27 Aug 2009; Barcelona; 2009.

[4] Hill WG, Zhang XS (2004). Effects on phenotypic variability of directional selection arising through genetic differences in residual variability. Genet Res.

[5] Mulder HA, Bijma P, Hill WG (2007). Prediction of breeding values and selection responses with genetic heterogeneity of environmental variance. Genetics.

[6] SanCristobal-Gaudy M, Bodin L, Elsen JM, Chevalet C (2001). Genetic components of litter size variability in sheep. Genet Sel Evol.

[7] Rönnegård L, Felleki M, Fikse F, Mulder HA, Strandberg E (2010). Genetic heterogeneity of residual variance—estimation of variance components using double hierarchical generalized linear models. Genet Sel Evol.

[8] Sorensen DA, Andersen S, Gianola D, Korsgaard I (1995). Bayesian inference in threshold models using Gibbs sampling. Genet Sel Evol.

[9] Ibáñez-Escriche N, Garcia M, Sorensen D (2010). GSEVM v.2: MCMC software to analyze genetically structured environmental variance models. J Anim Breed Genet.

[10] Perez-Cabal MA, Campos G, Vazquez AI, Gianola D, Rosa GJ, Weigel KA (2009). Genetic evaluation of susceptibility to clinical mastitis in Spanish Holstein cows. J Dairy Sci.

[11] Vazquez AI, Gianola D, Bates D, Weigel KA, Heringstad B (2009). Assessment of Poisson, logit, and linear models for genetic analysis of clinical mastitis in Norwegian Red cows. J Dairy Sci.

[12] Gianola D (1982). Theory and analysis of threshold characters. J Anim Sci.

[13] Ibáñez-Escriche N, Garcia M, Sorensen D (2010). GSEVM v. 3: MCMC software to analyse genetically structured environmental variance models. J Anim Breed Genet.

[14] Misztal I, Gianola D, Foulley JL (1989). Computing aspects of a nonlinear method of sire evaluation for categorical data. J Dairy Sci.

[15] Janssens S, Vandepitte W, Bodin L (2004). Genetic parameters for litter size in sheep: natural versus hormone-induced oestrus. Genet Sel Evol.

[16] Jousseins C, Trocmé E, Delmas D, Ducourtieux C, Mouret J, Dugat J, et al. Les coûts de production en élevage ovin allaitant dans le Sud-ouest—Campagne 2010. Réseaux d‘élevage pour le conseil et la prospective, collection référence. Paris: Institut de l’Élevage; 2012,

[17] Gilmour AR, Gogel BJ, Cullis BR, Thompson R (2009). ASReml user guide release 3.0.

[18] Schwarz G (1978). Estimating the dimension of a model. Ann Stat.

[19] Foulley JL, Gianola D (1996). Statistical analysis of ordered categorical data via a structural heteroskedastic threshold model. Genet Sel Evol.

[20] Hazel LN (1943). The genetic basis for constructing selection indexes. Genetics.

[21] Amer PR, Bodin L (2006). Quantitative genetic selection for twinning rate in ewes. Proc N Z Soc Anim Prod.

[22] Scheiner SM, Lyman RF (1991). The genetics of phenotypic plasticity. II. Response to selection. J Evol Biol.

[23] Matos CA, Thomas DL, Gianola D, Tempelman RJ, Young LD (1997). Genetic analysis of discrete reproductive traits in sheep using linear and nonlinear models: I. Estimation of genetic parameters. J Anim Sci.

[24] Janssens S, Vandepitte W, Bodin L (2004). Genetic parameters for litter size in sheep: natural versus hormone-induced oestrus. Genet Sel Evol.

[25] Matika O, van Wyk JB, Erasmus GJ, Baker RL (2003). Genetic parameter estimates in Sabi sheep. Livest Prod Sci.

[26] Garreau H, Bolet G, Larzul C, Robert-Granié C, Saleil G, SanCristobal M (2008). Results of four generations of a canalising selection for rabbit birth weight. Livest Sci.

[27] Gutiérrez JP, Nieto B, Piqueras P, Ibáñez N, Salgado C (2006). Genetic parameters for canalisation analysis of litter size and litter weight traits at birth in mice. Genet Sel Evol.

[28] Ros M, Sorensen D, Waagepetersen R, Dupont-Nivet M, SanCristobal M, Bonnet JC (2004). Evidence for genetic control of adult weight plasticity in the snail *Helix aspersa*. Genetics.

[29] Rowe SJ, White IM, Avendaño S, Hill WG (2006). Genetic heterogeneity of residual variance in broiler chickens. Genet Sel Evol.

[30] Sorensen D, Waagepetersen R (2003). Normal linear models with genetically structured residual variance heterogeneity: a case study. Genet Res.

[31] Ibáñez-Escriche N, Moreno A, Nieto B, Piqueras P, Salgado C, Gutiérrez JP (2008). Genetic parameters related to environmental variability of weight traits in a selection experiment for weight gain in mice; signs of correlated canalised response. Genet Sel Evol.

[32] Yang Y, Christensen OF, Sorensen D (2011). Analysis of a genetically structured variance heterogeneity model using the Box–Cox transformation. Genet Res (Camb).

[33] Martin P, Raoul J, Bodin L (2014). Effects of the *FecL* major gene in the Lacaune meat sheep population. Genet Sel Evol.

[34] Bodin L, Elsen JM (1989). Variability of litter size of French sheep breeds following natural or induced ovulation. Anim Sci.

